# Aspirin locally disrupts the liquid-ordered phase

**DOI:** 10.1098/rsos.171710

**Published:** 2018-02-28

**Authors:** Richard J. Alsop, Sebastian Himbert, Alexander Dhaliwal, Karin Schmalzl, Maikel C. Rheinstädter

**Affiliations:** 1 Department of Physics and Astronomy, McMaster University, Hamilton, Ontario, Canada; 2 JCNS, Forschungszentrum Jülich GmbH, Jülich Centre for Neutron Science at ILL, Grenoble, France

**Keywords:** lipid membranes, cholesterol, aspirin, liquid-ordered phase, membrane–drug interactions

## Abstract

Local structure and dynamics of lipid membranes play an important role in membrane function. The diffusion of small molecules, the curvature of lipids around a protein and the existence of cholesterol-rich lipid domains (rafts) are examples for the membrane to serve as a functional interface. The collective fluctuations of lipid tails, in particular, are relevant for diffusion of membrane constituents and small molecules in and across membranes, and for structure and formation of membrane domains. We studied the effect of aspirin (acetylsalicylic acid, ASA) on local structure and dynamics of membranes composed of dimyristoylphosphocholine (DMPC) and cholesterol. Aspirin is a common analgesic, but is also used in the treatment of cholesterol. Using coherent inelastic neutron scattering experiments and molecular dynamics (MD) simulations, we present evidence that ASA binds to liquid-ordered, raft-like domains and disturbs domain organization and dampens collective fluctuations. By hydrogen-bonding to lipid molecules, ASA forms ‘superfluid’ complexes with lipid molecules that can organize laterally in superlattices and suppress cholesterol’s ordering effect.

## 1. Introduction

Membrane research progresses with ever-increasing levels of granularity. Initial models of the membrane as an inert, physical barrier were revised with the discovery of membrane-embedded proteins. Singer & Nicholson [1] devised their famous ‘fluid mosaic’ model to describe a system where proteins float in a featureless soup of various lipid types. Most recently, membrane research has focused on membrane details on the level of individual lipids and proteins. The lipid environment in the specific region around a protein, such as the hydrophobic thickness or spontaneous curvature of the lipid tails, is now believed to be crucial to proper protein function [2]. Lateral diffusion of membrane constituents [3–7] and transmembrane diffusion of small molecules are believed to involve the local, collective motion of lipid tails [8–11].

Lipid rafts are an example of function arising from nanoscale structure. Rafts are small lipid heterogeneities in plasma membranes [12,13]. Rafts are typically described as structures that are enriched in cholesterol and exist to chaperone proteins from the Golgi apparatus to the plasma membrane surface [14]. Although their existence in biological membranes is still a matter of debate [15], various raft-like structures have been observed in model membranes, and from studies on model membranes, plasma membrane rafts are believed to be manifestations of the so-called liquid-ordered (*l*_o_) phase [12,16–27]. The *l*_o_ phase is unique in that it is characterized by elevated cholesterol concentrations as well as high lipid positional and chain order, however, at the same time low viscosity [28] and high surface tension. It is soft and stiff at the same time by being more rigid than a gel-state membrane, but less viscous than a fluid membrane.

The effect of drug molecules on lipid membranes is typically characterized by their effect on bulk membrane properties, such as mechanical properties and area per lipid head group [29–31], which affect, for instance, permeability. Drug–membrane interactions can also indirectly influence the function of membrane proteins through their membrane effects, and they also affect membrane heterogeneities [30,32,33]. For example, there are numerous reports that the common analgesic aspirin (acetylsalicylic acid, ASA) interacts with the lipid membrane and makes it softer and more fluid, and also impacts the formation of lipid raft structures [34–39]. Neutron scattering experiments recently presented evidence that aspirin creates local structural distortions in the *l*_o_ phase in model membranes [30], suppressing the formation of cholesterol clustering.

In this paper, we performed inelastic neutron scattering experiments and molecular dynamics (MD) simulations on membranes containing cholesterol and aspirin. The cholesterol concentration was chosen such that cholesterol rafts form, but well below the solubility limit of cholesterol [40]. We observed that aspirin creates local increases in area per lipid, causing a decrease in positional order and a damping of collective lipid tail fluctuations. We find evidence for ‘superfluid’ ASA–lipid complexes that organize in the membranes and impact cholesterol’s effect on the bilayers. We also present evidence for a direct ASA–cholesterol interaction in membranes through hydrogen bonding of ASA to cholesterol molecules.

## 2. Results

### 2.1. Inelastic neutron scattering

Coherent, inelastic neutron scattering experiments were performed on oriented DMPC-d54 (1,2-dimyristoyl-sn-glycero-3-phosphocholine) bilayers containing cholesterol and ASA. The lipid chains were selectively deuterated, enhancing the contribution of collective tail dynamics to the signal. In addition, the bilayers were hydrated with D_2_O to reduce incoherent contributions. Two membrane systems were prepared: bilayers with 32.5 mol% cholesterol (CHOL sample), and bilayers with 29 mol% cholesterol and 10 mol% ASA (ASA sample). This cholesterol concentration has been shown to form rafts before and is well below the solubility limit of cholesterol in dimyristoylphosphocholine (DMPC) of approximately 40 mol% [40]. Membranes were prepared by dissolving DMPC, cholesterol and aspirin in a 1 : 1 solution of trifluoroethanol/chloroform, at the appropriate molar ratios, and depositing the solution on 1×1 cm^2^ silicon wafers. Following drying in vacuum and incubation at 100% relative humidity for 48 h, highly oriented bilayers are formed [30,34,36,41]. Twenty such wafers were prepared and aligned with respect to each other to create a ‘sandwich sample’ with a total mosaicity of less than 0.5° and a total mass (lipids, cholesterol and aspirin) of 34 mg.

**Figure 1.**
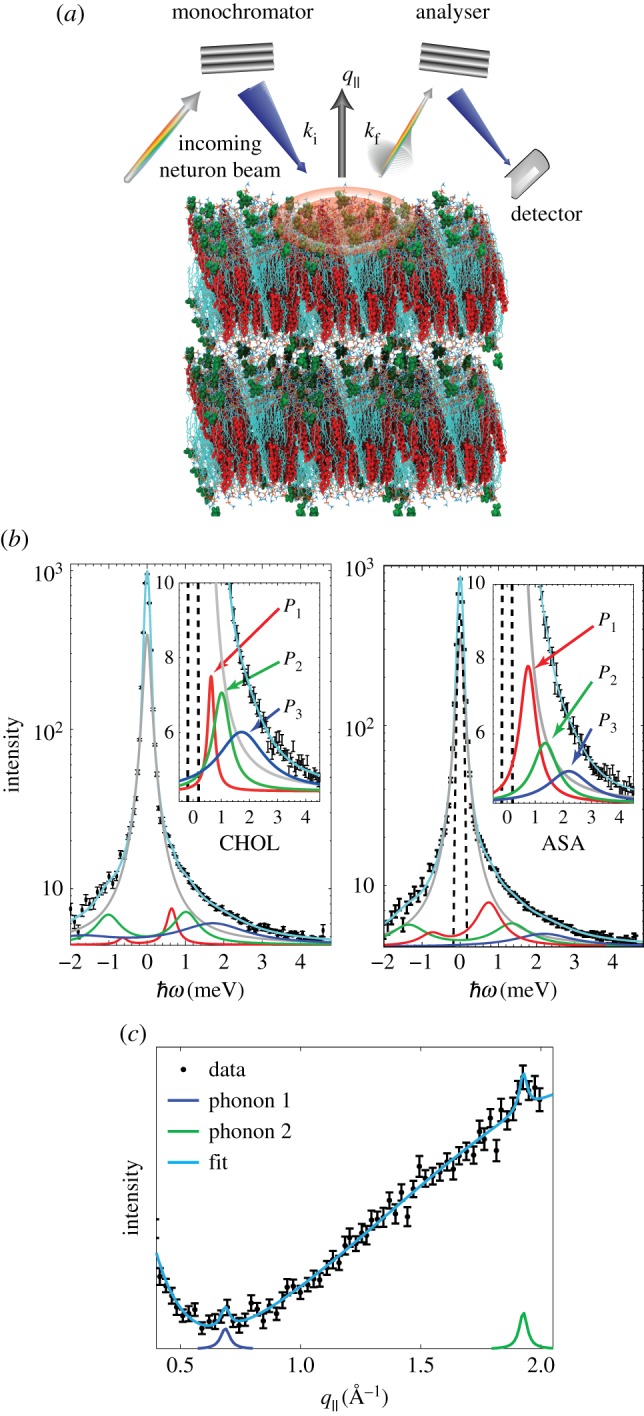
(*a*) Sketch of the scattering geometry. (*b*) Sample constant-*q*_∥_ scans for the CHOL sample at *q*_∥_=1.37 Å ^−1^. Data were fit using a Gaussian instrumental resolution, a Lorentzian peak centred at *q*_∥_=0 to capture incoherent scattering contributions, and three phonon excitations. The Gaussian instrumental resolution was fixed to values based on resolutions calculated using the ResLib package. The position of the incoherent peak was fixed at ℏω=0 meV; all other parameters are free. The inset shows three phonon excitations (P_1_, P_2_ and P_3_) in more detail. (*c*) Constant-energy scan for the ASA sample at an energy of ℏω=2.5 meV. All parameters are free in the constant-energy scans.

Neutron measurements were performed on the IN12 cold triple-axis spectrometer at the high flux reactor of the Institut Laue-Langevin (ILL) in Grenoble, France. All experiments were conducted at a temperature of 30°*C*, in the fluid phase of the membranes. The scattering vector ***Q*** was placed in the plane of the membranes (*q*_∥_) to measure the static (*S*(*q*_∥_)) and dynamic structure factors (*S*(*q*_∥_,*ω*)). A sketch of the scattering geometry is shown in figure 1*a*.

We note that IN12 was recently upgraded and was used in focusing mode, which significantly increases the neutron intensity at the sample position, however, at the cost of ***Q*** and energy resolution. When comparing our results to previous experiments in similar systems by Armstrong *et al.* [42] using a parallel beam configuration on IN12, signals in ***Q*** and energy, thus, appear slightly broader. In previous experiments, however, 400 mg of material was needed to conduct inelastic experiments, while only 34 mg was sufficient to see a clear signal in this paper.

Typical constant-*q*_∥_ scans, taken from the ASA and CHOL samples at *q*_∥_=1.37 Å ^−1^ are shown in figure 1*b*. The constant-*q*_∥_ scans were well fit by analytical functions described by three phonon excitations, as described in the Material and methods.

**Figure 2.**
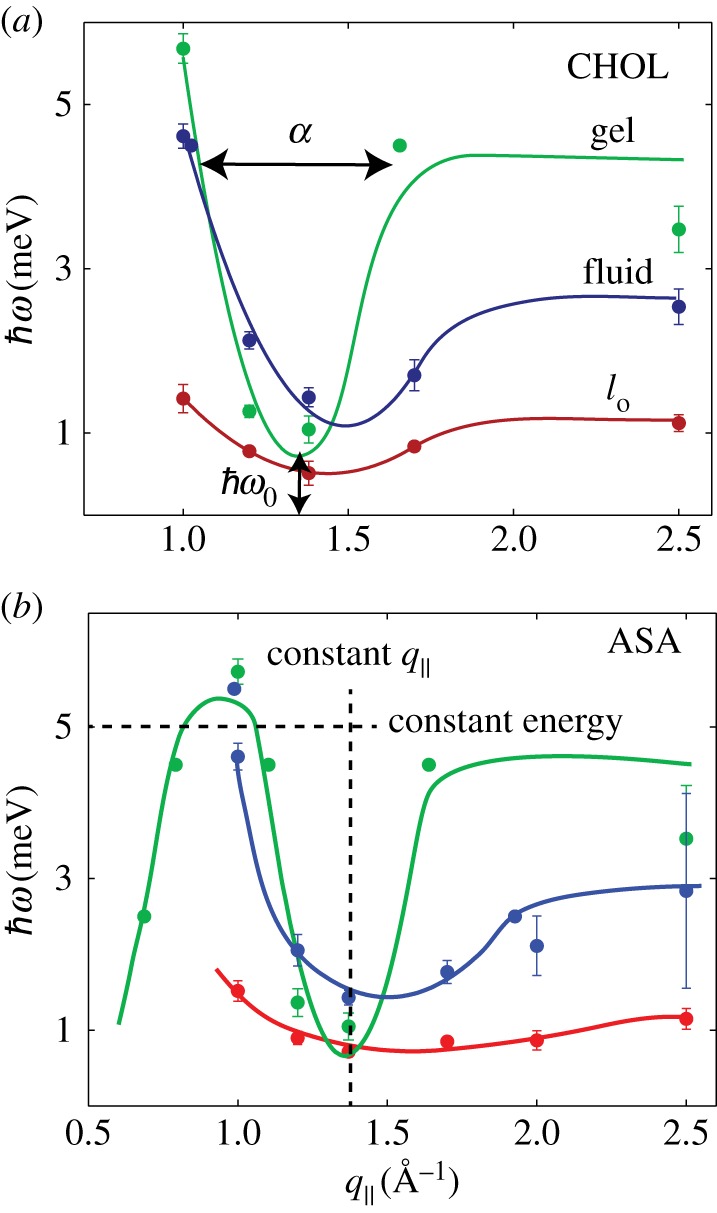
Dispersion relations for (*a*) the CHOL sample and (*b*) the ASA sample, as determined from constant-*q*_∥_ and constant-energy scans in figure 1. Three dispersion branches were observed, related to lipid molecules in gel (*P*_*β*_), fluid (*L*_*α*_) and liquid-ordered (*l*_o_) patches. The CHOL dispersion in (*a*) is in qualitative agreement with previous reports on the collective short-wavelength dynamics in cholesterol-rich lipid membranes [42]. The meaning of the parameters *α* and ℏω in equation (2.1) are displayed in (*a*). The direction of constant-*q*_∥_ and constant-energy scans is shown in (*b*).

A constant-energy scan at an energy of 2.5 meV is shown in figure 1*c* as an example. Constant-*E* scans were fit with an empirical exponential background, as well as a broad background Lorentzian peak at *q*_∥_∼2.5 Å ^−1^. The background peak arises from convolution of dynamics at higher *q*_∥_ values (figure 10). The position of the phonon branches are determined by the positions of the Lorentzian signals. The positions of phonons observed in both constant-*q*_∥_ and constant-*E* scans were combined to determine entire dispersion relations, shown in figure 2.

Lipid dispersions have been measured previously in inelastic X-ray and neutron scattering experiments [9–11,42–47] and using MD simulations [46,48,49]. All dispersions show similar features: at low *q*_∥_, the dispersion increases linearly with the slope related to the speed of sound. After reaching a maximum, the dispersion descends into a minimum at *q*_∥_=*q*_T_, where *q*_T_ typically agrees well with the position of the chain correlation peak (the maximum in the static structure factor). Finally, the dispersion increases with increasing *q*_∥_ to form a plateau.

The dispersion in the region of the minima can empirically be described by a quadratic function [42]:

2.1 $$\hbar \omega =\alpha {\left(q_{∥}-q_{∥0}\right)}^{2}+\hbar \omega_{0},$$

where $\hbar \omega_{0}$ is the minimum energy in the dispersion and *α* is the width of the dispersion; $\hbar \omega_{0}$ is related to lipid order, as a lower value indicates better-ordered, more ‘crystal-like’ bilayer; *α* is an empirical measure of the ‘softness’ of the bilayer [42].

As previously observed by Armstrong *et al.* [42], DMPC membranes containing cholesterol generate collective excitations belonging to three dispersions, related to coexisting gel, fluid and *l*_o_ phases. Evidence for this phase coexistence was also observed in the long wavelength fluctuations from neutron spin-echo measurements [28]. Armstrong *et al.* and Toppozini *et al.* later used neutron diffraction and demonstrated that *l*_o_ phases coexist in DMPC and DPPC bilayers with cholesterol [26,27,42,50,51].

Phonons were, therefore, assigned to three different phases. In the CHOL membranes shown in figure 2*a*, the gel phase (green) reaches ℏω>5 meV at *q*_∥_=1 Å ^−1^, then decreases to an energy minimum of ℏω∼0.7 meV at *q*_∥_∼1.4 Å ^−1^. A second of the phases is observed, with a wider minimum at ℏω∼1.1 meV (blue), and is assigned to the fluid phase. Finally, a third dispersion is recorded at lower energies, with a minimum ℏω∼0.6 meV. Armstrong *et al.* assigned the dispersion at low energy, which is only observed in the presence of cholesterol, to the *l*_o_ phase (red). The dispersion relations in the presence of ASA are shown in figure 2*b*.

**Figure 3.**
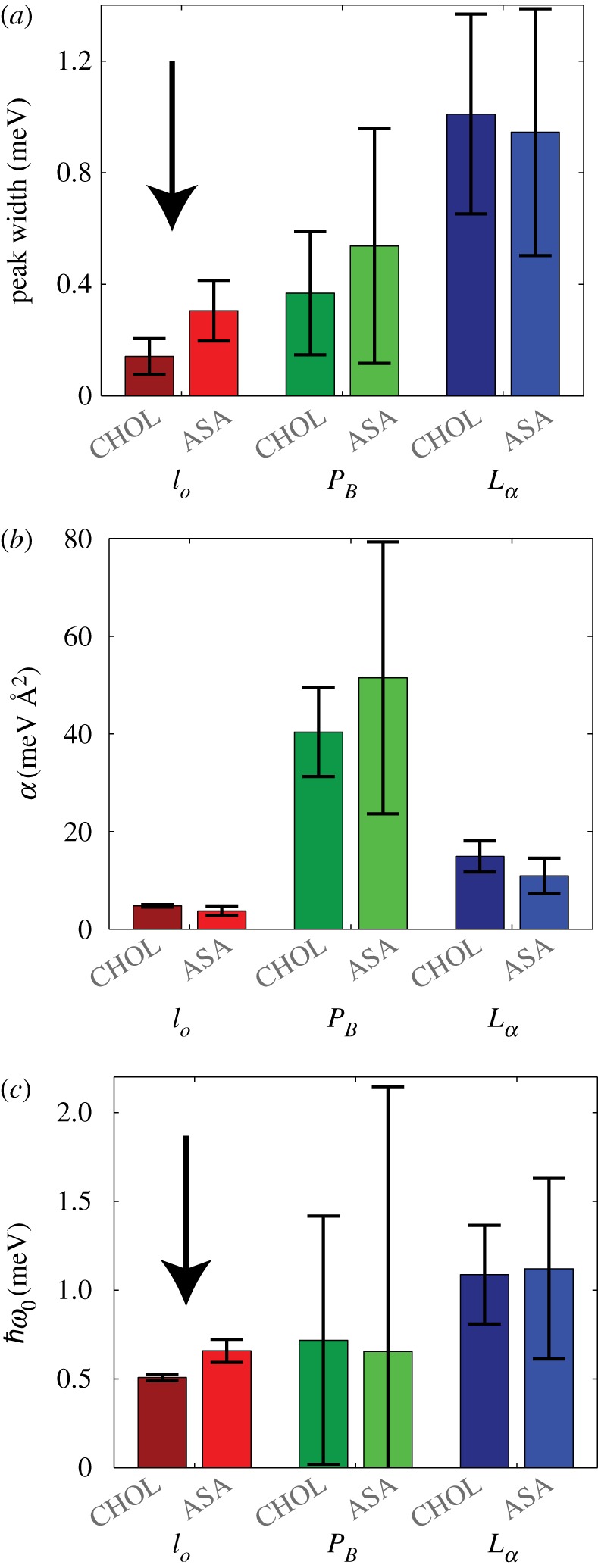
(*a*) Phonon widths for gel, fluid and *l*_o_ phase in figure 2 from phonon lines measured at *q*_∥_=1.37 Å ^−1^. (*b*) Parameters *α* and ℏω from equation (2.1) from fits to the dispersion curves. Statistically significant differences between CHOL and ASA samples were observed in the phonon width (*a*) and energy value of the minimum (*c*) in the *l*_o_ phase, only. Statistically significant results are indicated by the arrow.

With the phonons assigned to specific phases, the effect of ASA on the collective motions of each phase is examined. First of all, the damping of the phonons can be ascertained from the width of the phonon peak. As described in the Materials and methods, measurements along *q*_∥_=1.37 Å ^−1^ represent the truest representation of the phonon widths. An increase in peak width, from approximately 0.14 meV to approximately 0.31 meV, in the *l*_o_ phase is observed in the ASA sample. However, no statistically significant changes to the damping of the fluid or gel phases were observed, as displayed in figure 3*a*).

Equation (2.1) was fit to the phonon branches in the region 1.0 Å ^−1^<*q*_∥_<2.0 Å ^−1^, around the minimum. The fitting parameters are summarized in table 1 and also plotted in figure 3*b*,*c*. When comparing the dispersions from the ASA sample to the CHOL sample, two distinct points become important. First of all, for all structures, there is no statistically significant difference in bilayer softness, *α*. Secondly, we observe an increase in $\hbar \omega_{0}$ in the *l*_o_ phase for the ASA sample, indicating a decrease in lipid order. The increase in phonon damping and the decrease in lipid order are compatible with the occurrence of defects in the structure and a fluidification of the *l*_o_ phase, as will be discussed below.

**Table 1.** Results of the fits to equation (2.1). We see that *α* is a metric of the ‘stiffness’ of the tails on nanoscale distances; *ω*_0_ can be related to the order in the bilayer. Results are compared to Armstrong *et al.* [42] for DMPC membranes containing 40 mol% cholesterol. Parameters for the CHOL and ASA samples are also plotted in figure 3.

	*α* (meV/$q_{∥}^{2}$)			$\hbar \omega_{0}$ (meV)		
dispersion	Armstrong *et al.* (40 mol% CHOL)	cholesterol 30 mol%	ASA 10 mol%	Armstrong *et al.* (40 mol% CHOL)	cholesterol 30 mol%	ASA 10 mol%
gel	30.3	41 ± 9	51.5 ± 28	1.80	0.72 ± 0.70	0.65 ± 1.49
fluid	10.7	14.9 ± 3	11.0 ± 4	2.63	1.09 ± 0.28	1.12 ± 0.51
*l*_o_	6.6	4.8 ± 0.3	3.7 ± 0.9	1.09	0.51 ± 0.02	0.66 ± 0.07

### 2.2. Molecular dynamics simulation

To gain an atomistic view of aspirin’s effect on the *l*_o_ phase, MD simulations of membranes with 30 mol% cholesterol were performed. Simulations were performed on MacSim, a GPU-accelerated workstation, using parameters described previously [52] and also in the Material and methods. A united-atom DMPC + 30 mol% cholesterol bilayer, with 142 lipid + cholesterol molecules, was obtained from Hub *et al.* [53]. The number of water molecules per lipid was adjusted to 25, to mimic typical full-hydration conditions in agreement with the experiment, and the system was simulated for 200 ns. The membranes equilibrated to an area per lipid (DMPC and cholesterol) of *A*_*L*_=41.1 Å ^2^, which agrees closely with simulations by Hub *et al.* [53] and the experimentally determined area from Armstrong *et al.* [50]. In addition, the area per DMPC was determined using the GridMAT protocol to be 48.6±1 Å ^2^ [54], slightly smaller than the experimentally determined partial lipid area in membranes containing 32.5 mol% cholesterol [50].

**Figure 4.**
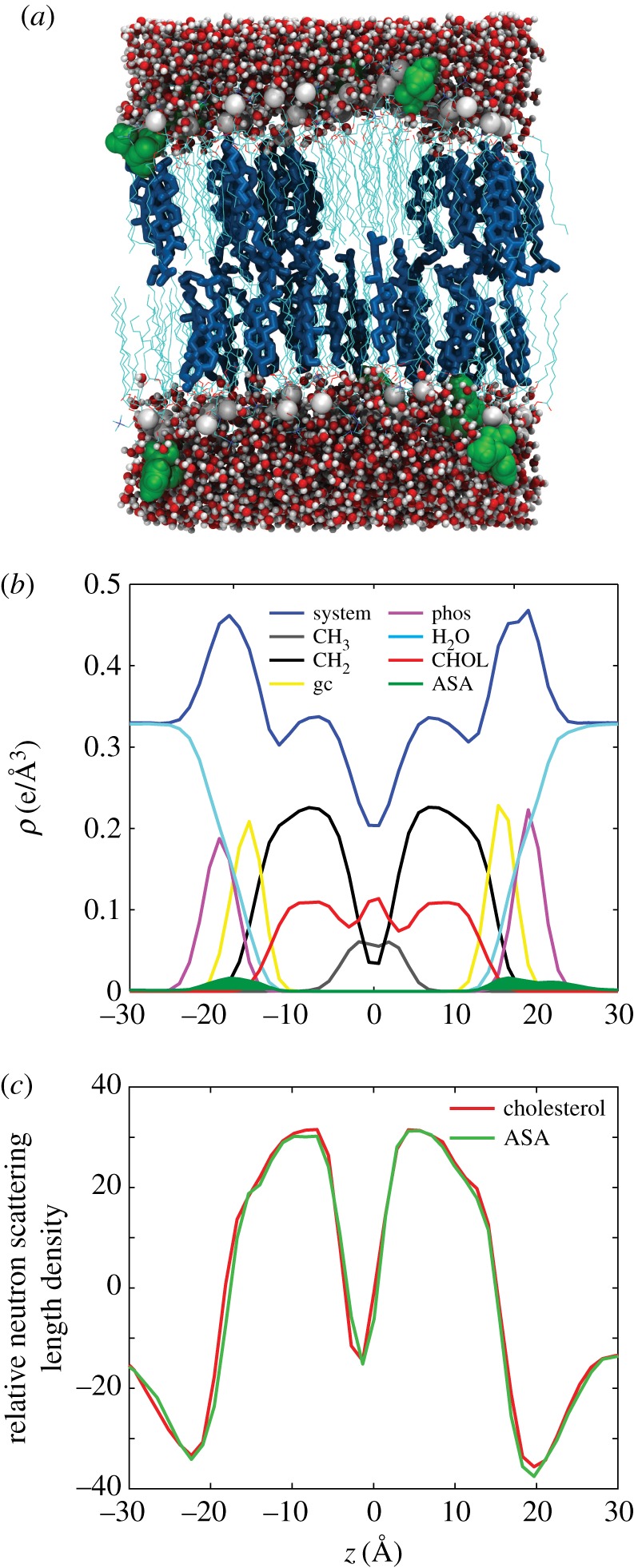
Results of the MD simulations. Simulations were performed for 30 mol% cholesterol in DMPC (CHOL), and the same system with 10 mol% ASA (ASA). (*a*) A snapshot of the ASA simulation. (*b*) An electron density breakdown for the components of the bilayer, indicating that the position of ASA (green) is in the head groups at |*z*|∼20 Å . (*c*) Neutron SLD from MD simulations. While the simulations were run in protonated membranes, the deuterium-labelled atoms were considered to be deuterated for the SLD calculation, as reported in [55].

Afterwards, the water in the system was removed and the aspirin was added to the now-dehydrated aqueous phase. Next, all water molecules (25 water molecules per lipid) were replaced and the system was equilibrated using the identical procedure as the pure bilayer. The equilibrated bilayer + aspirin system was then simulated for 200 ns. A snapshot of the simulation is shown in figure 4*a*. The molecules spontaneously embedded in the membrane within 10 ns. The density decomposition of the bilayer with ASA in figure 4*b* revealed that the molecules partitioned into the lipid head groups at *z*-values of |*z*|∼20 Å , in excellent agreement with past experiments [34,36]. To compare the MD simulations with the experiment, figure 4*c* shows the neutron scattering length density (SLD) with and without ASA. While the simulations were run in protonated membranes, the deuterium-labelled atoms were considered to be deuterated for the SLD calculation, as reported in [55]. The neutron scattering length agrees well with the experimentally determined neutron SLD shown figure 8*c*.

**Figure 5.**
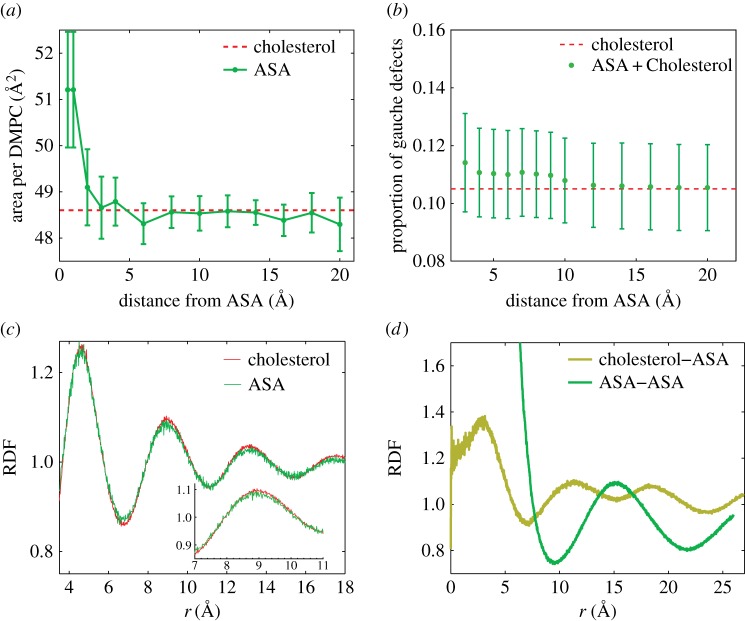
(*a*) Area per DMPC molecule at increasing distance from an ASA molecule. (*b*) Proportion of gauche defects in the DMPC molecules at increasing distance from ASA. (*c*) Radial distribution function (RDF) for DMPC–DMPC correlations, taken from both CHOL and ASA simulations. (*d*) RDF for cholesterol–ASA and ASA–ASA interactions.

A local change in the area per lipid near ASA molecules was observed. As depicted in figure 5*a*, DMPC molecules within an approximately 5 Å radius around an ASA molecule have an area per lipid of more than 50 Å ^2^, a 5% increase. As the area per lipid is a common proxy for membrane fluidity [56], these results suggest a local increase in fluidity due to ASA. The proportion of gauche defects in the lipid chains was calculated in figure 5*b*. The presence of ASA did not result in a statistically significant change in the number of tail gauche defects. So while ASA was found to increase *positional disorder*, the proportion of gauche defects in the lipid tails was found to be unchanged, indicating that ASA did not influence *chain segmental order* [57].

Radial distribution functions (RDFs) were calculated for DMPC–DMPC correlations. The RDF in figure 5*c* is proportional to the probability of finding a DMPC tail at a distance *r* from another DMPC tail. A peak at *r*∼4.8 Å indicates a lipid tail separation of 4.8 Å, in agreement with diffraction experiments in bilayer systems [42]. The lipid–lipid distance appears to be unchanged in the presence of ASA.

The simulations can in particular be used to search for correlations between cholesterol and ASA and ASA–ASA molecules. The cholesterol–ASA RDF is plotted in figure 5*d*. This function probes potential organization between cholesterol and ASA molecules in the bilayers. Peaks appear at *r*∼11.5 Å and 19 Å, indicating a preferred separation between ASA and cholesterol. Peaks are observed at length scales larger than a lipid–lipid distance, suggesting superlattice ordering, as has been suggested previously [30]. This ordering is further supported by the ASA–ASA RDF, also plotted in figure 5*d*. A maximum is observed at 15 Å as is a second peak just outside the measurement frame at 25 Å. Both peaks are compatible with a superlattice of lipid–ASA complexes, as will be discussed in the next section.

We note that an additional peak in the cholesterol–ASA RDF at |*r*|∼3 Å was observed. This was an important finding as, on visual inspection of the simulation results, ASA molecules were found to transiently hydrogen-bond with cholesterol molecules, without embedding in the bilayer.

## 3. Discussion

Much literature exists showing that ASA has a disordering effect on membranes [30,34,37,58]. This disordering effect is, for instance, used to explain how aspirin disrupts proteins embedded in lipid rafts [59]. So far, studies of drug–membrane interactions have mainly focused on bulk, global changes in membrane properties due to drugs, such as changes in phase behaviour (a decrease or broadening in the gel–fluid transition), or an overall change in bilayer thickness. However, physiological membranes are dynamic and heterogeneous, and their nanoscale properties, such as lipid curvature near a protein or the presence of domains, matter to the membrane’s function. In this paper, we used neutron scattering and MD simulations to demonstrate that ASA preferably interacts with the cholesterol-rich *l*_o_ phase and leads to local changes in the lipid arrangement and collective fluctuations.

ASA is known to partition into the lipid head groups and decrease lamellar spacing and widen the main transition in calorimetry experiments. Dispersion curves of the collective short-wavelength dynamics were measured in DMPC bilayers containing cholesterol and cholesterol and ASA. As was reported previously, the inelastic spectra present evidence for three dispersion branches, corresponding to lipids in their gel, fluid and liquid-ordered phase. By fitting an empirical function to the dispersion minimum, the degree of order and the softness of the corresponding phases were estimated from the depth of the minimum ($\hbar \omega_{0}$) and the width of the dispersion in the minimum (*α*). A statistically significant difference was observed in the energy value of the minimum. The increase in $\hbar \omega_{0}$ in the presence of ASA was assigned to a decrease in lipid order when ASA was present in the membranes. The decrease in order was accompanied by an increase in phonon line width, indicative of an increased phonon damping, probably related to the formation of defects.

Our experiments, therefore, present evidence that aspirin has the largest effect on *l*_o_ phases, where it reduces the size of coherently coupled patches [4] and makes the *l*_o_ phase overall more fluid. These observations are compatible with the idea that ASA forms superfluid complexes with lipid molecules that act as defects in the otherwise well-ordered *l*_o_ phase, as has been proposed by Alsop *et al.* using neutron diffraction [30]. It has been suggested that these complexes can organize throughout the *l*_o_ and form a superlattice-type structure in membranes with cholesterol and aspirin. The lattice spacing of this two-dimensional structure was reported as *a*=21.2 Å , *b*=18 Å , *γ*=103°. In addition, lipid–aspirin complexes were observed where aspirin caused local increases in the area per lipid. The authors proposed that ASA interacted with the *l*_o_ phase to create fluid patches and that these patches organize in the membranes and suppress cholesterol raft formation.

In experiments using highly monochromatic beams, such as synchrotron X-rays, local structure much smaller than the coherence length will be averaged with the surrounding membrane [27,50,51,60]. In a membrane at high coherence length, non-raft regions dominate in a diffraction measurement. By shrinking the coherence length of the X-ray or neutron probe, the scattering signal becomes an incoherent sum of many smaller coherent averages, giving more weight to the smaller scale structure. The longitudinal coherence length of a neutron beam, *ξ*, is defined by *ξ*=λ^2^/Δλ [61]; *ξ* has been estimated as $\xi =\sqrt{E}/\Delta E$ [50], where *E* is the energy of the neutron beam, and Δ*E* the energy resolution. In a typically monochromatic diffraction experiment, Δ*E* is small, making *ξ* large. Alsop *et al.* used a set-up with a low beam monochromaticity in their diffraction experiments to observe small, local structures in membranes with ASA and cholesterol with a coherence length, *ξ*, in the order of approximately 30 Å . The coherence length in our *inelastic* experiments is estimated as $\xi =18\sqrt{5}/1.5∼30\;\text{Å}$ (with the energy of the incident neutrons of approx. 5 meV measuring an excitation at approx. 1.5 meV). This value is comparable to the above diffraction experiments and also other inelastic neutron experiments in membranes [4]. We, therefore, argue that inelastic neutron scattering experiments can provide information about local structures in membranes.

**Figure 6.**
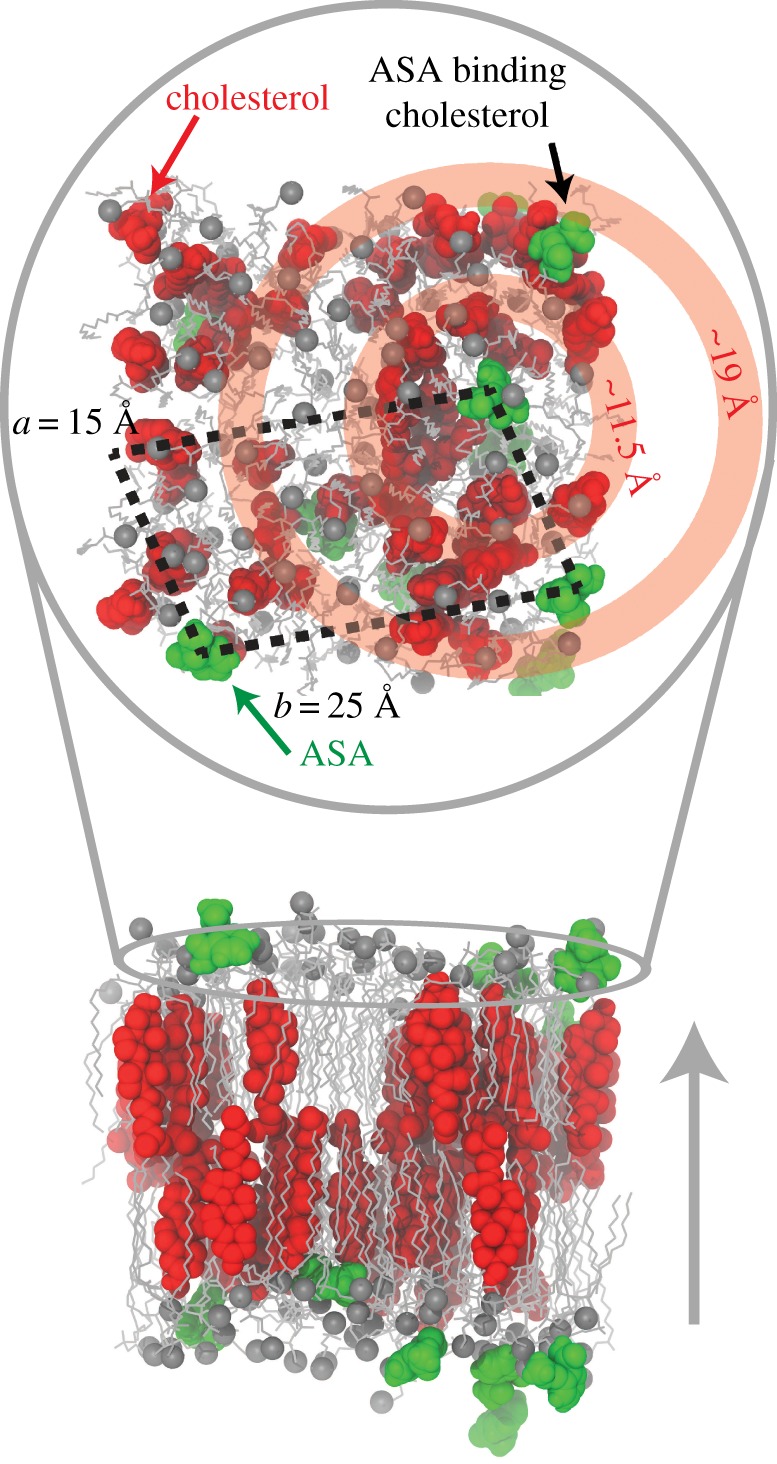
A simulation snapshot that captures the main points of the paper. The upper panel is an overhead view (along *q*_*z*_). The black dashed lines indicate the lateral separation of ASA into a lattice-like structure. The concentric red circles, centred on ASA, illustrate that cholesterol molecules prefer to be at regular distances from ASA.

Details of the molecular structure are assessed using our MD simulations. Lipid molecules in close proximity to ASA molecules were found to be more fluid, showing an increasing area per lipid. ASA and cholesterol molecules are surprisingly well organized, with well-defined nearest-neighbour distances. A snapshot of the structure of the membranes containing cholesterol and ASA from MD simulations is shown in figure 6. Here, the ASA–lipid complexes indeed form superlattices in the membrane plane with dimensions of 15 Å ×25 Å , in very good agreement with the experimentally determined dimensions. The peaks in the cholesterol–ASA RDF at 11.5 Å and 19 Å are roughly positioned at the minima in the ASA–ASA RDF, indicating that the cholesterol prefers to be in the lattice at positions not occupied by ASA.

An interesting question is why aspirin seems to be preferably attracted to *l*_o_ phases. It has been shown that ASA increases the solubility of cholesterol in lipid membranes [36] and has an effect on cholesterol-rich lipid rafts [30]. Aspirin’s affinity to *l*_o_ phases has recently also been demonstrated in human red blood cell plasma membranes [38]. This affinity could be entropy-driven, as ASA increases the fluidity of membranes and enhances lipid tail fluctuations. However, our results point to a free energy argument as the MD simulations also show evidence for ASA molecules hydrogen-bonding to cholesterol molecules. To the best of our knowledge, this is the first report of a direct interaction between ASA and cholesterol molecules in lipid bilayers.

## 4. Conclusion

By combining coherent, inelastic neutron scattering experiments and MD simulations, we investigated the effect of ASA on the liquid-ordered phase in phospholipid membranes. ASA was found to preferably bind to the liquid-ordered, raft-like domains. The dynamical experiments present evidence that ASA makes the *l*_o_ phase more fluid by creating local defects. From MD simulations, there is evidence that the ASA molecules form superfluid complexes by hydrogen-bonding to lipid molecules, which organize laterally in superlattices and suppress cholesterol’s ordering effect. There is also evidence for ASA molecules directly hydrogen bonding to cholesterol molecules in the bilayers. Our results are in agreement with previous observations in the literature and provide a molecular mode of action for aspirin’s known effect on membrane cholesterol.

## 5. Material and methods

### 5.1. Membrane preparation

Highly oriented multi-lamellar stacks of 1,2-dimyristoyl-sn-glycero-3-phosphocholine (DMPC), cholesterol and ASA were prepared on 1×1 cm, 300 μm thick, single-side polished Si wafers. The coherent scattering of the lipid hydrocarbon chains was enhanced by using tail-deuterated DMPC-d54. A 20 mg ml^−1^ solution of DMPC-d54 1 : 1 in chloroform and 2,2,2-trifluoroethanol (TFE) was prepared. Solutions of chloroform and ASA were also prepared in TFE : chloroform. DMPC, cholesterol and ASA were then mixed to achieve the desired molecular concentrations.

The Si wafers were cleaned with 30 min sonications in dichloromethane (DCM) at 310 K to remove all organic contamination and leave the substrates in a hydrophobic state. The wafers were then thoroughly rinsed three times using alternating approximately 50 ml of ultrapure water and methanol. The cleaned wafers were placed on a heated sample preparation surface, which was kept at 40°C (313 K). This temperature is above the main phase transition of DMPC, thus the heated substrates ensured that the lipids were in the fluid phase during deposition and the self-assembly of the lipids. An 80 μl aliquot of the lipid solution was deposited on each Si wafer in a tilting incubator, which was set to a speed of 15 rev min^−1^ and tilt of 10°, such that the lipid solution spread evenly across the wafer. The temperature was kept at 313 K and the solvent was allowed to slowly evaporate for 10 min. The wafers were kept in vacuum overnight to remove all traces of the solvent and then incubated with heavy water, D_2_O, at 313 K for 48 h. Following this protocol, each wafer contained approximately 3000 highly oriented membranes totalling approximately 10 μm in thickness.

**Figure 7.**
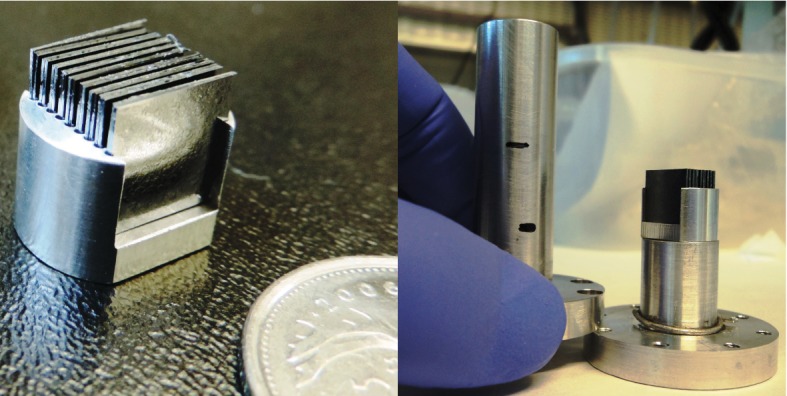
Photographs of the silicon wafers with highly oriented membranes. Wafers were mounted in the sample holder, custom manufactured for the IN12 cryostat. The water reservoir in the bottom of the can was filled with D_2_O to ensure hydration of the membranes during the elastic and inelastic scans. Sample can was sealed and mounted in an orange cryostat for temperature control (±0.01°C).

Eighteen sample-containing Si wafers were mounted in an aluminium sample holder fabricated to be inserted into IN12’s orange cryostat. A photo of the sample and the aluminium sample holder is shown in figure 7. Hydration of the lipid membranes from the vapour phase was achieved by a D_2_O reservoir in the bottom of the sample can. The samples were mounted vertically in the neutron beam such that the scattering vector (**Q**) could either be placed in the plane of the membrane (*q*_∥_), or perpendicular to the membrane (*q*_*z*_).

### 5.2. Neutron scattering

Experiments were conducted using the cold triple-axis spectrometer IN12 at the high flux reactor at the Institut Laue-Langevin in Grenoble, France. Two instrumental set-ups were used: (i) a lower resolution set-up with *k*_*f*_=1.5 Å ^−1^ and λ=4.2 Å , resulting in an energy resolution of approximately 0.3 meV and (ii) a higher resolution set-up with *k*_*f*_=1.1 Å ^−1^ and λ=5.71 Å , resulting in an energy resolution of approximately 0.2 meV . The higher resolution set-up was implemented at *q*_∥_=1.37 Å ^−1^ to verify the presence of three phonon peaks. The three axes of the spectrometers refer to the axes of rotation of the monochromator, the sample and the analyser. The incident and final neutron energies are defined by the Bragg reflections from pyrolytic graphite crystals. In-plane and out-of-plane structure can be measured simultaneously on a TAS by simply rotating the sample by 90°.

#### 5.2.1. Elastic scattering

**Figure 8.**
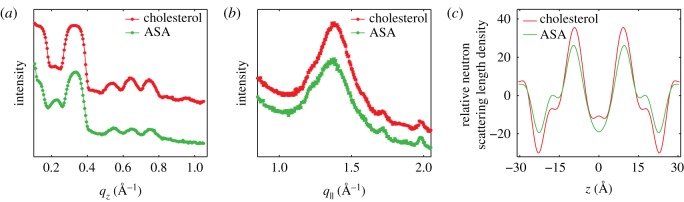
Results of elastic neutron scattering. (*a*) Out-of-plane neutron reflectivities along *q*_*z*_. Both the cholesterol and ASA samples show a set of approximately seven evenly spaced Bragg peaks. (*b*) In-plane elastic scattering along *q*_∥_. A broad feature is observed at *q*_∥_∼1.37 Å ^−1^. Additional reflections are associated with higher-order reflections of silicon. (*c*) Relative neutron SLDs assembled by Fourier analysis of the *q*_*z*_ peaks.

Elastic neutron scattering scans were taken along *q*_*z*_ (*q*_∥_=0) and *q*_∥_ (*q*_*z*_=0) to measure the static structure of the bilayers. Scans along *q*_*z*_ (figure 8*a*) observe a series of Bragg peaks, evenly separated by Δ*q*_*z*_, indicating well-ordered and lamellar samples. The lamellar spacing is calculated by *d*_*z*_=2*π*/Δ*q*_*z*_. We obtained *d*_*z*_=61.1 Å for the cholesterol sample and *d*_*z*_=60.6 Å for the ASA sample, which agrees with previous reports of a decrease in *d*_*z*_ for membrane samples with ASA [34,36] (table 2).

**Table 2.** Structural parameters determined from neutron diffraction.

sample	*d*_*z*_ (Å)	*d*_HH_ (Å)	*A*_T_ (Å^2^)
cholesterol	61.1	46.0	23.9
ASA	60.6	45	24.4

By integrating the lamellar peaks, the neutron SLDs, *ρ*_*z*_ are calculated using:

5.1 $$\rho_{z}=\sum_{n=1}^{N}\sqrt{I_{n}q_{n}}\nu_{n}\;\cos\;\left(\frac{2\pi z}{d_{z}}\right),$$

where *I*_*n*_ are the integrated peak intensities, *q*_*n*_ are the peak positions, and *ν*_*n*_ are the Fourier phases, which are ±1 for the stacked membrane systems. The phases for both samples are taken from Armstrong *et al.* as [1 −1 −1 −1 −1 1 1 −1 1] [42]. The resulting densities are shown in figure 8*c* on a relative scale. Unlike bilayer electron density profiles, neutron densities, for bilayers with deuterated chains, show a minima in the headgroups at |*z*|∼20 Å . The SLDs are maximum in the tails at |*z*|∼10 Å and at the edge of the bilayer, |*z*|=*d*_*z*_/2, where deuterated water and tails contribute. The position of the minima at |*z*|∼20 Å is used to determine the bilayer head–head spacing, *d*_HH_. In the cholesterol sample, *d*_HH_ was determined to *d*_HH_=46 Å and reduced to 45 Å for the ASA sample.

Elastic scans along *q*_∥_ measure the in-plane lipid structure, and are displayed in figure 8*b*. For both samples, a single intense peak is observed, associated with lipid tail correlations, at *q*_∥_∼1.37 Å ^−1^. Additional, smaller peaks are observed at *q*_∥_∼1.25 Å ^−1^, *q*_∥_∼1.7 Å ^−1^ and 1.95 Å ^−1^ associated with higher-order scattering of silicon and aluminium [30].

While the area per lipid is not directly accessible by in-plane scattering, the area per tail is calculated from *q*_T_ by $A_{T}=(8\pi^{2}/\sqrt{3})q_{T}^{2}$. *A*_T_=23.9 Å ^2^ for the cholesterol sample and *A*_T_=24.2 Å ^2^ for the ASA sample, a slight increase.

#### 5.2.2. Fitting the inelastic spectra

The constant-*q*_∥_ scans were well fit by analytical functions described by three contributions: the energy resolution of the spectrometer described by a Gaussian peak centred at ℏω=0 meV. The corresponding Gaussian width was calculated using the ResLib software using the geometry of the IN12 spectrometer [62]. Secondly, a Lorentzian peak, arising from incoherent molecular motions in the sample, centred at ℏω=0 meV. The remaining scattering contributions were fit by three phonon peaks, positioned at ℏω≠0. The width of these peaks is proportional to the damping experienced by the collective motions. The overall fitted analytic function also includes a linear background and can be written as

5.2 $$\begin{array}{rl} I(q_{∥}) & =A_{\mathrm{el}}\;\exp\;\left(-\frac{{\left(q_{∥}\right)}^{2}}{\left(2\sigma_{\mathrm{el}}^{2}\right)}\right)+mq_{∥}+b\\ & \;+\left[\sum_{i=1}^{3}\frac{A_{+,i}}{\left(1+{\left(q_{∥}-\mu_{i}\right)}^{2}/\sigma_{i}^{2}\right)}+\frac{A_{-,i}}{\left(1+{\left(q_{∥}+\mu_{i}\right)}^{2}/\sigma_{i}^{2}\right)}\right]+\frac{A_{\mathrm{incoherent}}}{\left(1+{\left(q_{∥}\right)}^{2}/\sigma_{\mathrm{inel}}^{2}\right)}. \end{array}$$

Fixed values were used for the position and width of the experimental resolution. The position of the incoherent peak was fixed to ℏω=0 meV. The ratio of the amplitudes *A*_+_ and *A*_−_ was determined by detailed balance.

**Figure 9.**
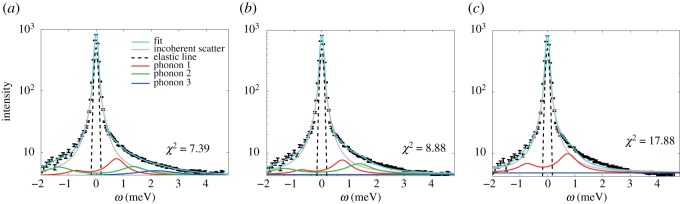
Inelastic scans measured at a *q*_∥_-value of *q*_∥_=1.37 Å ^−1^. The spectra can be fitted using different models: all models contain an elastic line, centred at energy transfer 0 (described by a Gaussian peak profile) and a Lorentzian peak also centred at ‘0’ to account for the incoherent scattering. (*a*) A fit including three phonon excitations, which shows a good agreement with the measured data. It was also attempted to fit the spectra with two excitations, and one excitation only (*c*). By comparing the resulting *χ*^2^-values, the fit with three phonons provides the best model to the data. This is also supported by the results of an *F*-test in table 3.

A comparison between fits using one, two and three phonon excitations is shown in figure 9. Based on the determined *χ*^2^-value, the three-phonon fit was found to best describe the experimental spectra. However, the inclusion of additional parameters into a fit function may lead to an improved fit without statistical significance. The quality of the model fit was, therefore, also studied using an *F*-test. The data at a *q*_∥_-value of *q*_∥_=1.37 Å ^−1^ were fitted using one, two and three phonon peaks successively. *χ*^2^ was determined for each fit as shown in figure 9. The *F*_*χ*_-value was calculated for every fit [63]:

5.3 $$F_{\chi }=\frac{\chi^{2}(m)-\chi^{2}(m+4)}{\chi^{2}(m+4)/(N-m-1)},$$

where m is the number of parameter used to fit *N* data points. The data included 108 data points. There are six parameters used to fit the background (two for the elastic peak, two for the inelastic peak and two for a linear background) and four parameters for every additional phonon signal. The calculated *F*_*χ*_-values are listed in table 3. According to [63] the additional parameters are valid if the *F*_*χ*_-value exceeds a critical *F*-value, which are listed in tables and were taken from [64]. Based on this test, the three-phonon fit indeed best describes the experimental data.

**Table 3.** *F*_*χ*_-values for for phonon fits when adding additional phonon peaks. The *F*_*χ*_-values increase by including two and eventually three-phonon excitations and exceed the *F*-value indicating that the inclusion of the additional parameters improves description of the experimental data. *F*-values were taken from database [64].

	*F*_*χ*_	*F*(2.5%)
adding second phonon	94.25	6.41
adding third phonon	17.94	6.04

#### 5.2.3. Resolution considerations

**Figure 10.**
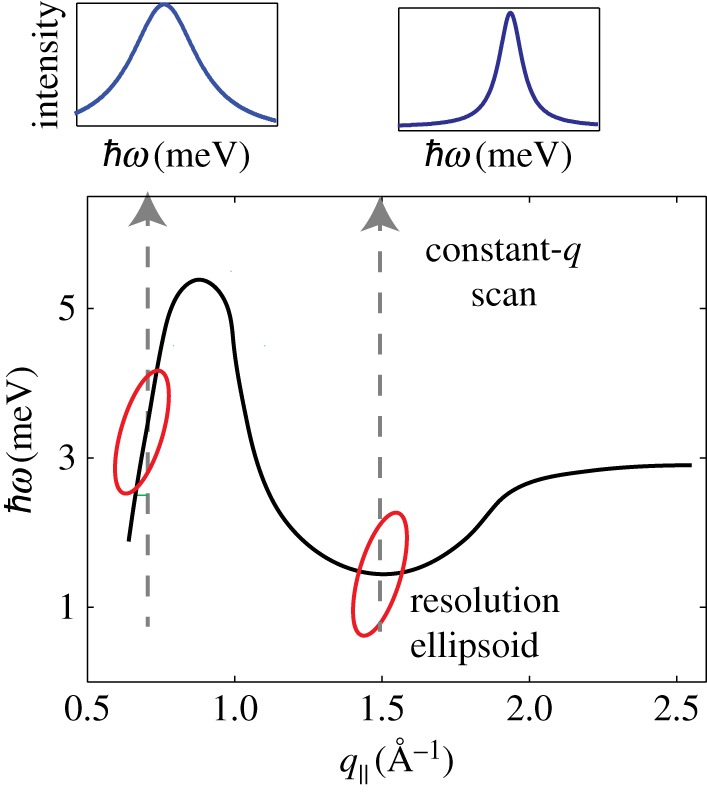
Lipid dispersion and instrumental resolution. The lipid dispersion is drawn schematically. During a constant-*q*_∥_ scan, the resolution ellipsoid is moved across the dispersion to detect the corresponding excitations. The smallest peak width in the spectra is observed when the dispersion is cut perpendicular, in the minimum of the dispersion relation.

The phonon peak width in the constant-*q*_∥_ energy scans is associated with phonon damping in the damped harmonic oscillator model. However, the width of the peak is not just a sample feature, but is convoluted with the instrumental resolution function. For a triple-axis spectrometer, this resolution function takes the form of a tilted elipsoid in the energy–momentum plane, where any excitation within the ellipsoid is recorded. A cartoon of the elipsoid on a lipid dispersion is in figure 10. When this ellipsoid intersects a region on the dispersion with high slope, the effect is to make peaks appear broader. Therefore, at the minima in the dispersion, where the slope is zero, a constant-*q*_∥_ scan would produce a peak width closest to the true width.

### 5.3. Molecular dynamics simulations

All simulations were run in-house on MacSim, a GPU-accelerated workstation with 20 physical Intel Xeon CPU cores and two GeForce GTX 1080 high-power graphics cards resulting in 5120 CUDA cores. This system produces about 180 ns per day of MD simulations in standard 128 lipid membrane patches in GROMACS.

A united-atom, DMPC + 30 mol% cholesterol bilayer with 142 lipid + cholesterol molecules, was obtained from Hub *et al.* [53]. Aspirin topology was obtained using the Automated Force Field Topology Builder (ATB) [65,66]. The SPC water model was used for system solvation [67]. All MD simulations were performed using the GROMACS 5.1.2 software package [68], implementing the GROMOS 54a7 force field [69] modified with Berger lipid parameters [70]. All simulations used a 2 fs time step, a periodic boundary condition applied to all directions, the particle-mesh Ewald to solve for long-range electrostatics [71], a short-range van der Waals cut-off of 1.2 nm and the LINCS algorithm to determine bond constraints [72]. Temperature coupling was controlled using a Nose-Hoover thermostat at 28°C (*τ*=0.5 ps) [73], and pressure was kept at 1.0 bar using Parrinello–Rahman semi-isotropic weak coupling (*τ*=1 ps) [74].

A total of two distinct simulations were conducted. First, the DMPC + cholesterol system was equilibrated with 25 waters per lipid for 200 ns. Next, the bilayer system with 10 mol% aspirin was prepared in a three-step process. First, all water in the system was removed, and the aspirin added to the now empty space outside the bilayer. Second, all water (25 per lipid) was replaced in the system. Finally, the system was re-equilibrated and then simulated for 200 ns. All analyses were performed with the final 50 ns of the simulations using GROMACS algorithms and simple scripts [75]. The electron density profiles were calculated for different constituents of the system. The function used calculates the relative distance along the bilayer normal of each atom within the specified index group, assigns a weighting based upon the number of electrons in each atom, and delivers an electron density as averaged over the specified time range.

The proportion of gauche dihedrals within a lipid system is commonly used as a measure of bilayer fluidity [76–78]. The proportion of gauche dihedrals as a function of increasing distance from aspirin was determined using dynamic scripting and GROMACS algorithms. A script was constructed to generate an index file containing only carbon chains belonging to lipids within the specified radius from the centre of mass of any aspirin molecule within the system every 50 frames. This index file specified the DMPC molecules whose carbons were to be used in calculation of the Ryckaert–Bellemans dihedral angles over that time interval. This was repeated over the final 50 ns of the simulation and averaged for each carbon position. Averaging across the SN1 and SN2 tails was then performed to generate the value shown in figure 5*d*, and the script was run successively to consider each new distance from aspirin.

## Supplementary Material

Inelastic Neutron scattering data

## Data accessibility

Inelastic neutron scattering data are provided as electronic supplementary material.
